# Association between safety climate, safety participation, safety compliance, and occupational injuries among workers in large-scale building construction projects in Ethiopia

**DOI:** 10.1007/s00420-025-02164-5

**Published:** 2025-08-30

**Authors:** Teferi Abegaz, Wakgari Deressa, Bente E. Moen

**Affiliations:** 1 Department of Preventive Medicine, School of Public Health, Addis Ababa University, P.O. Box 90861000, Addis Ababa, Ethiopia; 2 Department of Epidemiology and Biostatistics, School of Public Health, Addis Ababa University, P.O. Box 9086, Addis Ababa, Ethiopia; 3 Department of Global Public Health and Primary Care, Centre for International Health, University of Bergen, 5009 Bergen, Norway

**Keywords:** Safety climate, Safety performance, Safety behaviour, Building construction workers, Self-reported injury, Ethiopia

## Abstract

**Purpose:**

The construction industry is widely acknowledged as one of the most hazardous sectors for workers. This study examined the associations between safety climate and safety behaviour on self-reported injuries in large-scale construction sites in Ethiopia.

**Methods:**

A cross-sectional study was conducted from January to May 2023 among 1203 workers from 22 large-scale construction sites. Study participants from each site were selected using a proportional-to-the-size approach. The Nordic Safety Climate Questionnaire (NOSAQ-50) was administered using interviews. Binary logistic regression analysis was performed to identify the relationship between safety climate, safety behaviour, and other factors of self-reported injuries.

**Results:**

The prevalence of self-reported injuries in the last twelve months was 35.7% [95% CI (33.0, 38.4)]. Over one-third (35%) of the victims missed more than three workdays due to occupational injuries. Factors affecting self-reported injuries included being a carpenter [AOR = 2.86, 95% CI (1.91–4.28)], being an iron bender [AOR = 1.58, 95% CI (1.02–2.44)], having less than 5 years of work experience [AOR = 1.54, 95% CI (1.18–2.01)], lack of training [AOR = 2.16, 95% CI (1.27–3.72)], low safety climate [AOR = 1.53, 95% CI 1.06–2.21)], low safety participation [AOR = 2.16, 95% CI 1.64–2.86)], and low safety compliance [AOR = 2.32, 95% CI 1.79–3.02)].

**Conclusions:**

This study revealed a high magnitude of injuries and identified a relationship between safety climate, safety behaviors, and occupational injuries in the construction industry. Ensuring the work sites' safety climate and improving compliance with safety rules and procedures is essential.

## Introduction

The construction industry plays a pivotal role in global economic development. Yet, it is widely recognized as one of the most hazardous sectors for workers, with injuries and fatalities occurring at alarmingly high rates (Liao and Perng 2008; Mohamed et al. 2009). Workers in this sector often operate under constantly changing weather conditions (Rozenfeld et al. 2010) and navigate environments filled with movements of people, materials, and heavy machinery (Sacks et al. 2009). Tasks frequently require working at significant heights, such as on roofs, ladders, and scaffolding, often using poorly designed or malfunctioning equipment (Ajith et al. 2020; HSE 2020). Additionally, construction sites are complex workplaces where main contractors and sub-contractors often work together, leading to coordination challenges that can further increase safety risks (Kshaf et al. 2022).

Compared to other industries, the construction industry has higher rates of injuries, resulting in significant productivity losses, lifelong disabilities, and fatalities (Arndt et al. 2005; Juhari et al. 2023). These injuries not only lead to human loss but also incur substantial costs, delay project completion, and damage to the reputation of construction firms (Mahmoudi et al. 2014; Mustard and Yanar 2023). For instance, data from Great Britain shows that the accident rate in the construction sector is four times higher than the total number of accidents reported across all industries (HSE 2020). In the United States, construction-related accidents occur nearly three times more often than in other sectors (CCRT 2015). The burden of injury is even more challenging in developing countries, where work practices remain highly traditional and reliant on manual labor (Boadu et al. 2020; Wu et al. 2018). This global trend highlights the pressing need for improved safety management practices, particularly in low-income countries like Ethiopia.

In Ethiopia, the construction sector represents a significant public health challenge, with a large workforce operating in hazardous conditions (GBN 2020). However, the lack of a comprehensive accident recording and reporting system makes it difficult to understand the prevalence and causes of work-related injuries fully. A systematic review in Ethiopia reported a pooled prevalence of self-reported injuries in the construction industry at 45.6% (Meseret et al. 2022), underscoring the urgent need for better injury surveillance and prevention strategies in the construction sector.

Given the high risks associated with construction work, proactive management is essential. Research has highlighted the role of safety climate and safety behaviours in reducing injuries during construction works (DeArmond et al. 2011; Zahoor et al. 2017). Safety climate refers to workers’ shared perceptions of their organization’s commitment to safety and has been shown to influence safety outcomes in high-risk industries significantly (Christian et al. 2009; Hon et al. 2014). Safety participation involves voluntary actions that contribute to workplace safety, such as assisting coworkers with safety issues and attending safety meetings or training (Clarke 2006). However, safety compliance includes mandatory actions that every employee must follow to maintain minimum safety standards at work, such as using personal protective equipment (PPE) and adhering to safety protocols (Neal and Griffin 2006).

Despite the significant role of safety climate and safety behaviors in reducing injuries, no study has been conducted in Ethiopia to explore these factors in the construction sector. Therefore, this study aimed to investigate the relationship between safety climate, safety behaviors (including safety participation and compliance), and self-reported occupational injuries in Ethiopia's construction industry. The findings of this study will provide valuable insights into the prevalence of injuries and the role of safety management frameworks in the construction industry, and that this information can be used to improve workplace safety and reduce injuries.

## Methods and materials

### Study design and setting

This study employed a cross-sectional design conducted from January to May 2023 to investigate safety climate, safety behavior, and work-related injuries in 22 randomly selected large-scale construction sites across five major cities in Ethiopia. According to the official report from the Federal Construction Regulatory Authority, more than 120 large-scale construction sites were operating in the country during the study period. The cities included in the study were Addis Ababa, Adama, Bishoftu, Bahir Dar, and Hawassa, the cities were chosen for their high number of construction activities.

### Sample size and sampling procedures

The sample size was determined using a single population proportion formula, accounting for a design effect of 2 and a 10% non-response rate, resulting in a total sample of 1250 workers.

A two-stage sampling approach was employed. First, 22 construction sites were selected using proportional representation from the total 88 construction sites operated in the selected regions. Specifically, 12 sites were chosen from Addis Ababa, three from Adama, two from Bishoftu, three from Bahir Dar, and two from Hawassa, based on the distribution of construction sites across these cities. In the second stage, workers from each selected site were chosen using a proportional-to-the-population-size sampling method, ensuring representation from different departments, including managers/supervisors, skilled workers, and unskilled workers. A list of workers, including their respective departments, was provided by each company and used as a sampling frame. Workers employed at the company for at least one year were eligible to participate. This criterion was chosen to estimate the one-year prevalence of self-reported injury rates. Study participants were then selected using a proportional-to-the-size approach. In most cases, we used the list of workers as a sampling frame to select individual participants. However, on some sites, it was challenging to use the list, especially when certain workers on the list were reassigned to other sites owned by the same company during the data collection period. In those cases, we randomly selected participants without using the list.

### Data collection tools and procedures

Data were collected through face-to-face interviews using structured questionnaires. We also observe each construction site using an observational checklist. The Nordic Safety Climate Questionnaire (NOSACQ-50) was used to measure the safety climate conditions in the construction companies and translated into more than 35 languages including Amharic (Kines et al. 2011; Marin et al. 2019; Yousefi et al. 2016). This validated tool consists of seven safety climate dimensions, comprising 50 items. Of these, 22 items evaluate safety at the management level, and 28 items at the worker’s level. A four-point Likert Scale (1 = strongly disagree, 2 = disagree, 3 = agree, and 4 = strongly agree) was used for the answers in the questionnaire. To measure safety behaviour, a set of nine items was adopted from previous studies (Mohamed 2002; Nadhim et al. 2018; Neal and Griffin 2006). Five items were used to measure the respondents’ level of participation in safety activities. Likewise, four items were used to measure the level of safety compliance. A five-point Likert scale was adopted to measure the response to each item, ranging from 1 to 5, in terms of strongly disagree, disagree, neutral, agree, and strongly agree, respectively. The details of the tool used in this study are outlined in the forthcoming publication (Abegaz et al. 2025).

A questionnaire template was loaded onto each data collector's smartphone with an Open Data Kit (ODK) and 20 trained and experienced data collectors participated in the data collection process. Pre-testing was conducted in one large-scale real estate development project site located around CMC area in Addis Ababa, which is not part of the actual study sites. Based on the pre-test results, adjustments were made to the data collection manual to clarify certain questions. Each interview session took on average 15 to 20 min. The interviews were conducted in a private setting with only the worker and one interviewer present; no personal identification was recorded, and the information the workers provided was kept confidential. Two senior supervisors monitored the data collection process. To ensure reliability and standardization across multiple interviewers, we implemented several measures before and during data collection. We provide comprehensive training that includes instruction on the study protocol, use of the ODK platform, and standardized approaches to administering the questionnaire, which is supported by preparing a detailed data collection manual. The training also included role plays and a field pilot to reinforce consistency. Additionally, supervisors closely monitored data collection in the field, conducting regular spot checks.

### Data handling and statistical analysis

Data were analyzed using Statistical Package for the Social Sciences (SPSS) version 23 software (IBM Corp., Armonk, NY, USA). Data were checked for missing values, outliers, and inconsistencies. Descriptive statistics (frequencies, percentages, means, and cross-tabulations) were performed to summarize the data and the results were presented using tables and figures. The mean, standard deviation, and reliability of the safety climate and safety behaviour factors were calculated. The reliability coefficient for the safety climate and safety behavior scales was calculated using Cronbach’s alpha, with all dimensions showing reliability above 70%, indicating acceptable internal consistency. The multicollinearity was assessed using Variance Inflation Factors (VIF), and all the values were below the recommended level. Self-reported injuries were considered the dependent variable, while socio-demographic characteristics, company-related characteristics, work environment characteristics, safety climate, safety participation, and safety compliance were the independent variables. Bivariate and multivariate logistic regression analyses were performed using a binary logistic regression model. Odds ratios (OR) and 95% confidence intervals (CI) were reported, with a significance level set at *p* < 0.05.

Based on the Nationale Forskningscenter for Arbejdsmiljø (NFA) interpretation guideline of the NOSACQ-50 safety climate questionnaire, a score above 3.30 indicates a good safety climate in the workplace. Scores between 3.00 and 3.30 are considered fairly good, though some improvement is needed. Scores ranging from 2.70 to 2.99 are viewed as fairly low, indicating the need for improvement, while scores below 2.70 are considered low and signal a significant need for improvement (NFA 2017). Therefore, to make it more interpretable, we classified it into low levels (scores below 2.70 and scores ranging from 2.70 to 2.99) and good levels (scores ranging from 3.00 to 3.30 and scores above 3.30). We focused on the total safety climate score in this study.

Regarding safety participation and safety compliance, we interpreted the Likert scale weighted mean using the interval method for classification. This method divides the scale into specific intervals to assess the level of safety participation and compliance, providing a structured way to evaluate the responses based on the weighted mean score (Pimentel 2010). Accordingly, it is interpreted as strongly disagree (1.0–1.80), disagree (1.81–2.61), neutral (2.62–3.42), agree (3.43–4.23), and strongly agree (4.24–5.0). To make it more interpretable we classified it into low levels which include strongly disagree, disagree and neutral (1.0–3.42), and good levels include agree and strongly agree (3.43 – 5.0).

### Operational definitions

Large-scale building construction project: Building construction project with an estimated capital budget of above 100,000,000 Ethiopian Birr (ETB) which is nearly equivalent to $ 2 million.

Self-reported injury: Any physical injury during the work on the construction site that led to more than three days of absenteeism, two to three days of absenteeism, or required first aid without absenteeism, reported by the worker during the past year.

## Results

Overall, we approached 1250 randomly selected individuals, including site managers, safety engineers, supervisors, and other frontline construction workers. Of these, 1,203 participated in the study, yielding a 96.2% response rate, while 47 (3.8%) individuals declined to participate for various reasons. More than half (56.4%) of the participants were between the ages of 25 and 34 (Table 1). The mean age of study participants was 29.6 years (SD ± 6.9). Most participants were male (87.9%) and more than half (51.0%) were married. About (3.1%) of participants had no formal education, and a quarter (32.5%) attained tertiary education (Table 1).

**Table 1:** Socio-demographic and organizational characteristics of large-scale construction industry workers

Variable	Category	Frequency	Percentage
Age in years	18–24	264	21.9
	25–34	679	56.4
	35–44	211	17.5
	45–54	40	3.3
	> = 55	9	0.7
Sex	Male	1057	87.9
	Female	146	12.1
Marital status	Single	572	47.5
	Married	613	51.0
	Divorced/widowed	18	1.4
Educational status	No formal education	38	3.1
	Primary education	382	31.8
	Secondary education	392	32.6
	College diploma and above	391	32.5

About two-thirds (62.3%) of the participants were from Addis Ababa, while 84.2% were from local constructors (Table 2). The vast majority of participants (80.2%) were front-line construction workers. The mean work experience of workers in the construction industry was 6 years (SD + 4.9). Most (91.9%) study participants didn’t receive safety training last year. The mean score for all safety climate dimensions was 2.70.

**Table 2:** Workplace and behavioural characteristics among large-scale building construction workers

Variable	Category	Frequency	Percentage
Site location	Addis Ababa	749	62.3
	Amhara	151	12.6
	Oromia	153	12.7
	Sidama	150	12.5
Contractor type	Local	1013	84.2
	International	190	15.8
Job category	Site supervisor	238	19.8
	Mason	272	22.6
	Carpenters	203	16.9
	Iron benders	149	12.4
	Finishing	112	9.3
	Assistants/Others	229	19.0
Work experience	< = 5 years	736	61.2
	> 5 years	467	38,8
On-job safety training (last year)	No	1105	91.9
	Yes	98	8.1

As illustrated inTable 3,among safety behaviour dimensions the mean score of safety compliance (3.58) was higher than safety participation (2.95). Two-thirds (63.3%) of participants showed low participation in health and safety matters. More than one-third (35.0%) of the respondents showed low compliance with safety practices (following safety rules and using personal protective devices). The majority (84.4%) of workers perceived that the safety climate in the organization was low.

**Table 3:** Behavioural characteristics among large-scale building construction workers

Variable	Category	Frequency	Percentage
Safety participation	Good level	442	36.7
	Low level	761	63.3
Safety compliance	Good level	782	65.0
	Low level	421	35.0
Safety climate	Good level	188	15.6
	Low level	1015	84.4

Low levels (scores below 2.70 and scores ranging from 2.70 to 2.99) and good levels (scores ranging from 3.00 to 3.30 and scores above 3.30).

As shown in Table 4, around 36% [95% CI (33.0, 38.4)] of the participants reported occupational injuries in construction activity in the past 12 months. In the current analysis, injuries were classified into three categories: those resulting in more than three days of absenteeism, those resulting in one to three days of absence, and those resulting in no absenteeism, which accounts for (35.0%), (29.4%) and (35.6%), respectively. Most (83.9%) of injured participants reported being injured more than once in the past year.

**Table 4:** Injury characteristics and mechanisms among large-scale building construction workers

Variables	Response	Frequency	%
Injury (*N* = 1203)	Yes	429	35.7
	No	774	64.3
Injury categories (*N* = 429)	Injury of more than three days of absenteeism	150	35.0
	Injury of one to three days of absenteeism	279	29.4
	Injury without absenteeism	153	35.6
Frequency of injury (*N* = 429)	Once	360	83.9
	Twice	41	9.6
	More than twice	28	6.5
The pattern of activities during the accident (*N* = 429)	Actual task	389	90.7
	Movement/transit	40	9.3
Nature of injury (More than one option is possible) (*N* = 531)	Abrasion/laceration	176	33.1
	Dislocation/fracture	72	13.6
	Cut	242	45.6
	Eye injury	23	4.3
	Others	18	3.4
Body parts injured (More than one option is possible) (*N* = 656)	Head and neck	56	7.8
	Eye	23	8.5
	Upper extremities	142	21.6
	Lower extremities	305	46.5
	Chest and abdomen	119	18.1
	Others	11	1.7

**Fig. 1:**
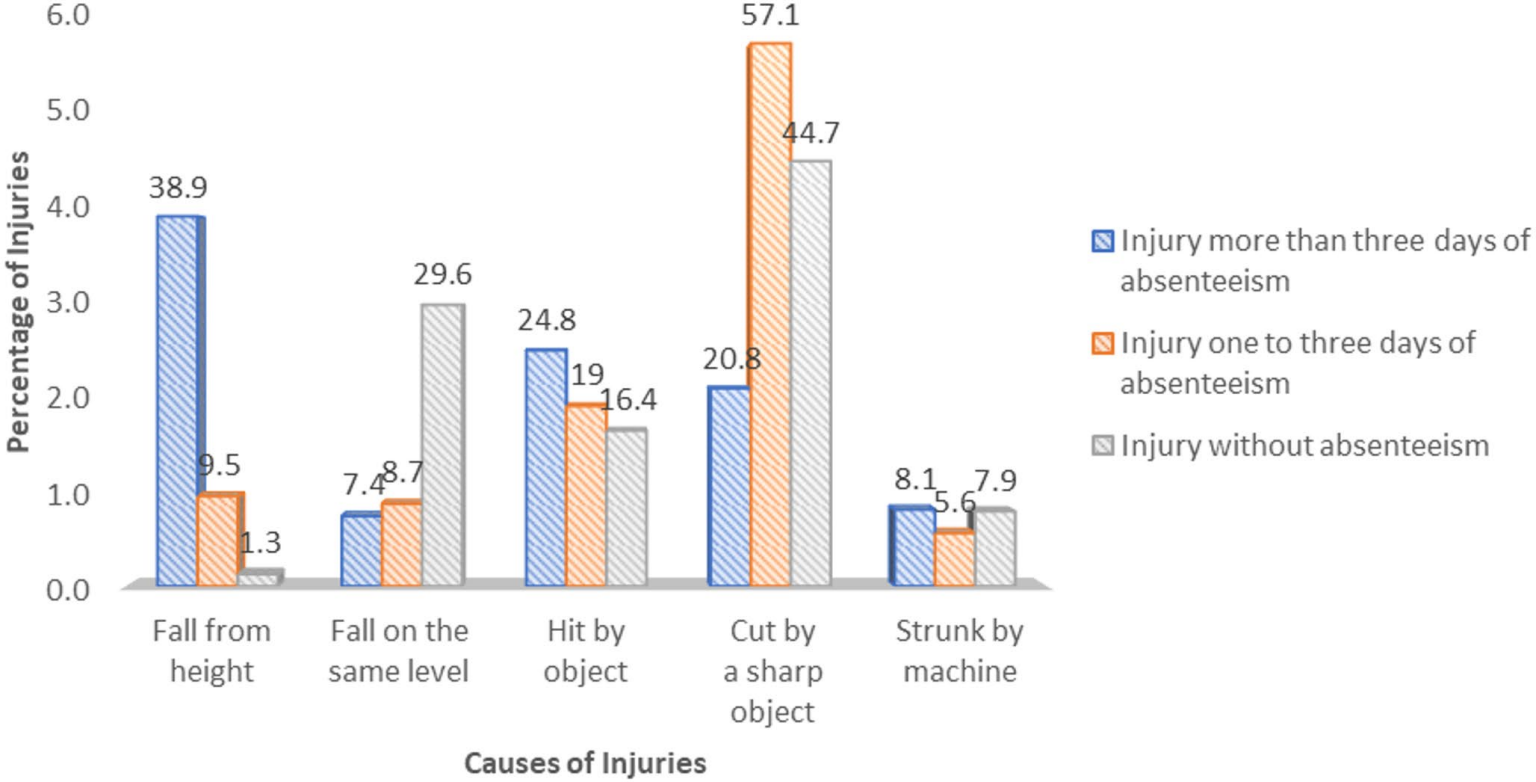
Mechanism of injuries among large-scale building construction workers

As illustrated in Fig. 1, falls from height were the primary cause (38.9%) of injuries for people who missed more than three days of work followed by heat by objects (24.8%). For those injuries resulting in one to three days of absence from work and injuries without absenteeism, the main injury mechanism was cut by sharp objects accounting for (57.1%) and (44.7%) respectively.

The multivariate logistic regression analysis revealed that seven factors were significantly associated with self-reported injury. Variables with a *p* value <  = 0.2 in the bivariate analysis were considered in the multivariate analysis. As shown in Table 5 construction sites operated by local contractors were 49% more likely to experience injuries than international contractors. Among all job categories, the odds of self-reported injuries were nearly three times higher among carpenters and 58% more among iron benders compared with site supervisory staff. Likewise, workers with less work experience (less than 5 years) in the construction industry experienced 54% higher odds of injury than workers with more than 5 years of experience. Those workers who didn’t receive job safety training in the last 12 months experienced injuries more than twice as often as those who received the training. Similarly, workers who perceived a low safety climate level had a 53% higher likelihood of self-reported injuries than those who reported a good safety climate level. Employees who did not actively participate in safety issues experienced injuries more than twice as often as those who participated in safety issues. Similarly, employees who did not comply with safety (disrespecting safety regulations and not wearing personal protective equipment) were injured more than twice as often as those who complied with safety.

**Table 5:** Multivariate analysis of factors associated with occupational injuries among large-scale building construction workers

Characteristics		Self-reported injury		COR (95%CI)*	AOR (95%CI)*
	Category	No	Yes		
Site location	Outside Addis Ababa	284 (62.6)	170 (37.4)	1.13 (0.89–1.44)	0.98 (0.74–1.30)
	Addis Ababa	490 (65.4	259 (34.6)	1	1
Constructor type	Local	637 (62.9)	376 (37.1)	1.53 (1.08–2.15)**	1.49 (1.02–2.18)*
	International	137 (72.1)	53 (27.9)	1	1
Job category	Mason	182 (66.9)	90 (33.1)	1.37 (0.94–2.02)	1.20 (0.81–1.76)
	Carpenters	93 (45.8)	110 (54.2)	3.29 (2.21–4.90)***	2.86 (1.91–4.28)***
	Iron benders	90 (60.4)	59 (39.6)	1.82 (1.18–2.82)***	1.58 (1.02–2.44)*
	Finishing	78 (69.6)	34 (30.4)	1.21 (0.74–1.99)	1.18 (0.71–1.97)
	Assistance/Others	156 (68.1)	73 (31.9)	1.30 (0.87–1.94)	1.24 (0.73–2.16)
	Site supervisory staffs	175 (73.5)	63 (26.5)	1	1
Work experience	< = 5 years	447 (60.7)	289 (39.3)	1.51 (1.18–1.93)***	1.54 (1.18–2.01)***
	> 5 years	327 (70.0)	140 (30.0)	1	1
On-job safety training	No	694 (62.8)	411 (37.2)	2.63 (1.56–4.45)***	2.16 (1.64–2.86)***
	Yes	80 (81.6)	18 (18.4)	1	1
Safety climate	Low level	634 (62.5)	381 (37.5)	1.75 (1.23–2.49)***	1.53 (1.06–2.21)***
	Good level	140 (74.5)	48 (25.5)	1	1
Safety participation	Low level	439 (57.7)	322 (42.3)	2.30 (1.77–2.98)***	2.16 (1.64–2.86)***
	Good level	335 (75.8)	107 (24.2)	1	1
Safety compliance	Low level	207 (49.2)	214 (50.8)	2.73 (2.13–3.49)***	2.32 (1.79–3.02)***
	Good level	567 (72.5)	215 (27.5)	1	

***P value < 0.001;**P value < 0.01; *P value < 0.5; COR: Crude odds ratio; AOR: Adjusted odds ratio

## Discussion

The prevalence of self-reported occupational injuries among large-scale building construction employees was reported to be 35.7% in the past year. Several factors were associated with self-reported injuries, including employment by domestic contractors, carpentry or iron/aluminum workers, inexperienced workers, lack of safety training, low safety climate, inadequate safety participation, and lack of safety compliance.

The prevalence of injuries in the present study was lower than the pooled prevalence of 45.6% reported in a meta-analysis of Ethiopian construction workers (Meseret et al. 2022). This difference may be attributed to our focus on large-scale construction sites, which often have better safety practices compared to smaller companies. Evidence shows that large-scale companies usually have better health and safety policies and practices than small-scale ones (ILO 2021). A scoping review has shown that employees in smaller construction companies are more vulnerable to injuries (Howe et al. 2024). Our findings align with a Ugandan study reporting a 32.4% prevalence (Kiconco et al. 2019), suggesting regional similarities in construction practices and safety challenges. In contrast, the prevalence reported in the present study was higher than the 20.4% reported in a Turkish study (Başağa et al. 2018), likely reflecting differences in labor intensity and technological support. In Ethiopia, construction activity is more labor intensive, with less technical support.

In the current study, the prevalence of self-reported injury is lower among international contractors compared to local contractors. It supports evidence from the Nigerian construction sector (Vacanas et al. 2020), which suggests that international contractors tend to have better safety performance due to their larger resources, stronger safety cultures, access to advanced technology, and experience working in various regulatory environments (Vacanas et al. 2020). This finding is also supported by the evidence collected through the observational checklist. The use of personal protective devices and the installation of safety measures while working at higher elevations is evident in all of the sites managed by the international contractors. Local contractors, while sometimes more familiar with the specific environmental conditions and local regulations, may not always have the same level of safety resources or training (Wubet et al. 2021). However, safety performance can also vary significantly within both groups, and individual contractors' commitment to safety is a key factor regardless of their size or location.

This study found that carpenters and iron binders as particularly vulnerable to injuries. These findings are consistent with studies in Russia (Timofeevaa et al. 2017) and Nigeria (Okoye 2018), which similarly highlight high-risk activities. In Ethiopia, it is common for carpenters to operate at heights where there is a considerable risk of falling. Evidence shows falls from height are one of the most common mechanisms of injury in the construction industry (Abukhashabah et al. 2019). Therefore, it is crucial to monitor the quality of scaffolding and ladders in the construction sites.

Research indicates the frequency of injuries on construction sites is significantly influenced by work experience. In our study, inexperienced workers were more likely to sustain injuries than more experienced ones. It supports a study conducted in northern Greece, where the majority of accidents involve inexperienced personnel (Sotiris Betsis et al. 2019). A study from the Polish construction industry shows longer work experience is associated with fewer accidents (Szóstak 2019). This could be a result of people learning from past mistakes and reducing their risk-taking behavior. Less experienced workers may lack the knowledge and awareness necessary to prevent hazards effectively, emphasizing the importance of mentorship and on-the-job training to complement formal instruction.

Occupational health and safety training plays a significant role in injury prevention. In the current study, workers who received training had significantly lower odds of injury, consistent with findings from Northeast Ethiopia (Gebremeskel and Yimer 2019) and a national scoping review (Ashuro et al. 2021; Meseret et al. 2022). Evidence shows that health and safety training helps employees to better understand the potential risk of occupational accidents, to follow work rules and procedures, and to use personal protective devices (Meseret et al. 2022).

In this study, a significant association was found between self-reported injury rates and the level of safety climate. This result is consistent with previous research, which shows that an unfavorable safety climate doubles the risk of injury (Min et al. 2024). A positive safety climate plays a crucial role in reducing construction injuries by influencing attitudes, behaviors, and practices that foster a safer work environment. When the safety climate is favorable workers are more likely to engage in safer behaviors, leading to fewer accidents (Hon et al. 2014; Mohammadfam et al. 2022). Similarly, when safety is emphasized within a company culture, workers are more likely to follow safety protocols, decreasing the risk of injuries and accidents and they also feel a sense of responsibility for both their safety and the safety of their colleagues (Erkal et al. 2021).

In the current study, safety participation was found to have a statistically significant association with injury. This finding is consistent with previous research indicating that safety participation has a delayed impact on accident involvement (Neal and Griffin 2006). When workers actively engage in safety training and meetings, they gain a better understanding of safety issues and are more likely to adopt safe behaviors (OSHA 2016). Safety participation is vital in construction injury prevention for several reasons. It not only safeguards workers but also contributes to the overall success of a construction project. When workers are actively involved in safety programs, they feel more connected to the project, becoming more vigilant and proactive in their work (Ajmal et al. 2022). This increased engagement leads to better job satisfaction and a reduced risk of injury. Workers who are actively involved in safety initiatives understand their role in the process and feel a sense of accountability for their actions and those of their peers (DeArmond et al. 2011). Overall, safety participation encourages workers to take the necessary precautions to avoid health risks, such as wearing protective gear.

Evidence demonstrates that safety compliance is essential in reducing construction injuries. In the current study, a statistically significant association was found between safety compliance and self-reported injury, which aligns with previous research (Al-Bayati et al. 2023). When employees adhere to safety protocols, such as wearing personal protective equipment (PPE) and following proper work procedures, the likelihood of accidents and their severity decreases (Ashuro et al. 2021; Sehsah et al. 2020). Safety compliance ensures that the work environment meets safety standards, identifies and mitigates hazards, promotes continuous improvement, and fosters a safety culture. By following these regulations, construction companies can reduce accidents, prevent injuries, and improve both worker well-being and project efficiency. Compliance with safety standards leads to enforcing critical safety protocols, such as fall protection, hazard communication, and lockout/tagout procedures.

The study was conducted in five cities across Ethiopia, a low-income country. Due to the random selection of representative locations, the findings are likely generalizable to the country. Furthermore, the results may be relevant to other low-income settings, particularly in East Africa, where similar economic conditions, construction practices, and occupational safety challenges exist. Additionally, the study's insights could apply to other low-income regions worldwide, offering valuable implications for improving workplace safety and reducing occupational injuries in comparable contexts.

### Limitations

Some limitations of this study should be noted. The use of cross-sectional studies captures data at a single point in time, so they cannot determine whether poor safety climate leads to more injuries or if experiencing injuries influences perceptions of safety climate (reverse causality). However, it provides a useful snapshot of the association between variables and helps identify potential associations that can guide future longitudinal research. The use of self-reported injury data might underestimate the actual number of occupational injuries due to recall bias. In addition, a healthy worker effect might be present, as the study did not include absent workers, who might have left the workplace due to an injury. To avoid the recall bias, we limit the reporting period to 12 months. To mitigate underreporting due to fear of employer retaliation, we emphasized confidentiality and anonymity throughout the data collection process. In addition, the use of self-reported data through questionnaires which are considered reliable and valid sources (Gabbe et al. 2003). In the current study, we classified injury severity based on workplace absenteeism can incur misclassification of the severity level of the injury. However, in the absence of clinical data and the low literacy level of the study participants, injury classification based on workplace absenteeism can objectively assess injury severity. Another possible limitation of self-reported research is the presence of social desirability bias, where employees may give more socially acceptable answers about their actual daily practices. However, Occupational injuries are concrete events rather than subjective opinions or behaviors, and social desirability bias may have a limited impact on the findings. The use of the NOSAQ-50 safety climate tool also minimizes such biases as it focuses more on group perceptions than on individual workers. Therefore, it might not have a serious implication in the present study. We dichotomized the NOSACQ-50 scores into “low” and “good” instead of using continuous or multilevel categorization, which might obscure the subtle differences in safety climate. We apply this categorization to facilitate interpretability and to align with previous literature.

## Conclusions

This study highlights a high prevalence of self-reported occupational injuries among workers in large-scale building construction sites in Ethiopia, Carpenters, iron benders, and inexperienced employees were found to be at higher risk of injury, emphasizing the need for targeted interventions. The findings underscore the importance of fostering a positive safety climate and promoting safety behaviors such as compliance with safety protocols and active participation in safety activities. Addressing these issues requires improving the safety climate, enforcing compliance with safety standards, and mandating regular safety training for all construction companies and their workers. These efforts are essential for reducing injuries in the construction sector.

Future research should explore the perspectives of key stakeholders to better understand and address safety challenges. A qualitative investigation of the perspectives of clients, consultants, contractors, and government representatives about safety is suggested. Future longitudinal or prospective studies are recommended to better establish temporal relationships and causality. We also recommended that future research integrate clinical severity measures alongside absenteeism to better capture the full spectrum of injury impact.

## Data Availability

The authors are willing to provide the raw data used in this study upon request.
